# Comparison of Myoelectric Control Schemes for Simultaneous Hand and Wrist Movement using Chronically Implanted Electromyography: A Case Series

**DOI:** 10.1109/EMBC46164.2021.9630845

**Published:** 2021-11

**Authors:** Jacob L. Segil, Platon Lukyanenko, Joris Lambrecht, Richard F. ff. Weir, Dustin Tyler

**Affiliations:** Rocky Mountain Regional VA Medical Center, Rehabilitation Research and Development, Denver, CO 80220, USA; Biomedical Engineering Program, University of Colorado Boulder, CO 80309, USA; Department of Biomedical Engineering, Case Western Reserve University, Cleveland OH, 44106 USA; APT Center, Louis Stokes Cleveland Veterans Affairs Medical Center, Cleveland, OH 44106, USA; Department of Biomedical Engineering, Case Western Reserve University, Cleveland OH, 44106 USA; FES Center, Louis Stokes Cleveland Veterans Affairs Medical Center, Cleveland, OH 44106, USA; Rocky Mountain Regional VA Medical Center, Rehabilitation Research and Development, Denver, CO 80220, USA; Department of Bioengineering, University of Colorado Denver|Anschutz Medical Campus, Aurora, CO, 80045, USA; Case Western Reserve University, Department of Biomedical Engineering, Cleveland, OH, 44106, USA; Louis Stokes Cleveland Veterans Affairs Medical Center, Cleveland, OH, 44106, USA

## Abstract

**Objective:**

A current biomedical engineering challenge is the development of a system that allows fluid control of multi-functional prosthetic devices through a human-machine interface. Here we probe this challenge by studying two subjects with trans-radial limb loss as they control a virtual hand and wrist system using 6 or 8 chronically implanted intramuscular electromyographic (iEMG) signals. The subjects successfully controlled a 4, 5, and 6 Degrees of Freedom (DoF’s) virtual hand and wrist systems to perform a target matching task.

**Approach:**

Two control systems were evaluated where one tied EMG features directly to movement directions (Direct Control) and the other method determines user intent in the context of prior training data (Linear Interpolation).

**Main Results:**

Subjects successfully matched most targets with both controllers but differences were seen as the complexity of the virtual limb system increased. The Direct Control method encountered difficulty due to crosstalk at higher DoF’s. The Linear Interpolation method reduced crosstalk effects and outperformed Direct Control at higher DoF’s. This work also studied the use of the Postural Control Algorithm to control the hand postures simultaneously with wrist degrees of freedom.

**Significance:**

This work presents preliminary evidence that the PC algorithm can be used in conjunction with wrist control, that Direct Control with iEMG signals allows stable 4-DoF control, and that EMG pre-processing using the Linear Interpolation method can improve performance at 5 and 6-DoF’s.

## I. Introduction

A significant short-coming in the field of upper limb prosthetics is the lack of a human-machine interface (HMI) capable of providing users with dexterous and simultaneous control over all Degrees of Freedom (DoF) provided by state-of-the-art prosthetic limbs [1]. Such systems include many independently controllable DoF’s including multi-functional prosthetic hands [2] and wrists [3]. In clinical settings multi-DoF systems are often controlled by adjusting each DoF sequentially. Sequential prosthesis motion is burdensome and slow compared to the simultaneous and natural motion provided by intact limbs [4]. Simultaneous motion can be enabled by improving the number and independence of control signals decoded from user activity or by developing controllers that decipher and command simultaneous movement.

Clinical prosthetic systems are typically controlled by body-powered interfaces or by myoelectric devices that decode user intent from surface electromyography (sEMG) [4]. Prior work with sEMG has shown some simultaneous control, but typically does not exceed three Degrees of Actuation (DoA) [5]–[10]. Here, DoA will refer to the dimensionality of the control signal sent to the prosthetic device. This is partially caused by the simultaneously recording activity from several muscles when using sEMG which complicates the process of decoding user intent. Implanted EMG shows reduced muscle ‘crosstalk’ [11], and can be collected with implantable myoelectric sensors [12]–[16] and regenerative peripheral nerve interfaces (RPNI, [17], [18]). Intramuscular EMG is presently suggested to be a preferred method for recording EMG for myoelectric control strategies [15], [19]–[21], and recently demonstrated simultaneous 4-DoA velocity control with chronically implanted EMG (iEMG) [16].

Movement simultaneity can also be increased by coupling prosthesis DoF (e.g. controlling grasps rather than joints). This allows a low-DoA controller to command a high-DoF device Here, DoF refers to the number of independent actuators within the electromechanical system. Prosthesis DoF can be coupled through the Postural Control (PC) Algorithm [22]–[25] and other methods [26], [27]. The PC algorithm was previously shown to work in 2-DoA / 4-DoF cases using sEMG with Direct Control [25], [28]. In Direct Control (DC), EMG sites are directly mapped to DoF directions after filtering, gains, and thresholds are applied. A DC-PC system does not force movement into discrete states or require extensive training procedures while allowing linear combinations of hand grasps to be reached in a proportional manner. To date, the advantages of such a system have just begun to be explored in take home studies [28].

In this study we examine three hypotheses in a velocity-control target-matching task with two subjects implanted with iEMG electrodes. First, we extend on past work that evaluated a 2-DoA/4-DoF DC-PC sEMG system [23], [24] by examining performance in iEMG systems ranging in complexity from 2-DoA/4-DoF to 4-DoA/6-DoF. We hypothesize that (1) iEMG systems will allow more complex control than past sEMG systems and that (2) performance will degrade as the number of DoA controlled increases even for iEMG based controllers. Second, we explore pre-processing techniques for improving control signal independence. In particular, we hypothesize that (3) the 4-DoA Linear Interpolation (LI) controller can combine with the PC algorithm to create a 4-DoA/6-DoF LI-PC iEMG system that will be superior to the DC-PC iEMG system.

## II. Methods

### A. Research Subjects

Two subjects with unilateral trans-radial limb loss participated in this study. Subject S8, the deidentified label for the subject, was previously implanted with 8 pairs of iEMG [29] electrodes interfacing with pronator teres (P), flexor carpi radialis (FCR), flexor digitorum superficialis (FDS), flexor carpi ulnaris (FCU), supinator (S), extensor carpi radialis longus (ECRL), extensor digitorum (ED), and extensor carpi ulnaris (ECU). Surgery details describing this implant type are provided by Dewald et al [15]. Subject S6 had similar implants, excluding the pronator teres and ECU. Both subjects had substantial experience with prior experiments using myoelectric control algorithms. All clinical research was performed under approved IRB protocol (IRB # 16050-H37) from the Louis Stokes Cleveland Department of Veterans Affairs Medical Center and under an active Food and Drug Administration (FDA) Investigational Device Exemption (IDE), G110043.

### B. Target Matching Task

**Figure 1–:**
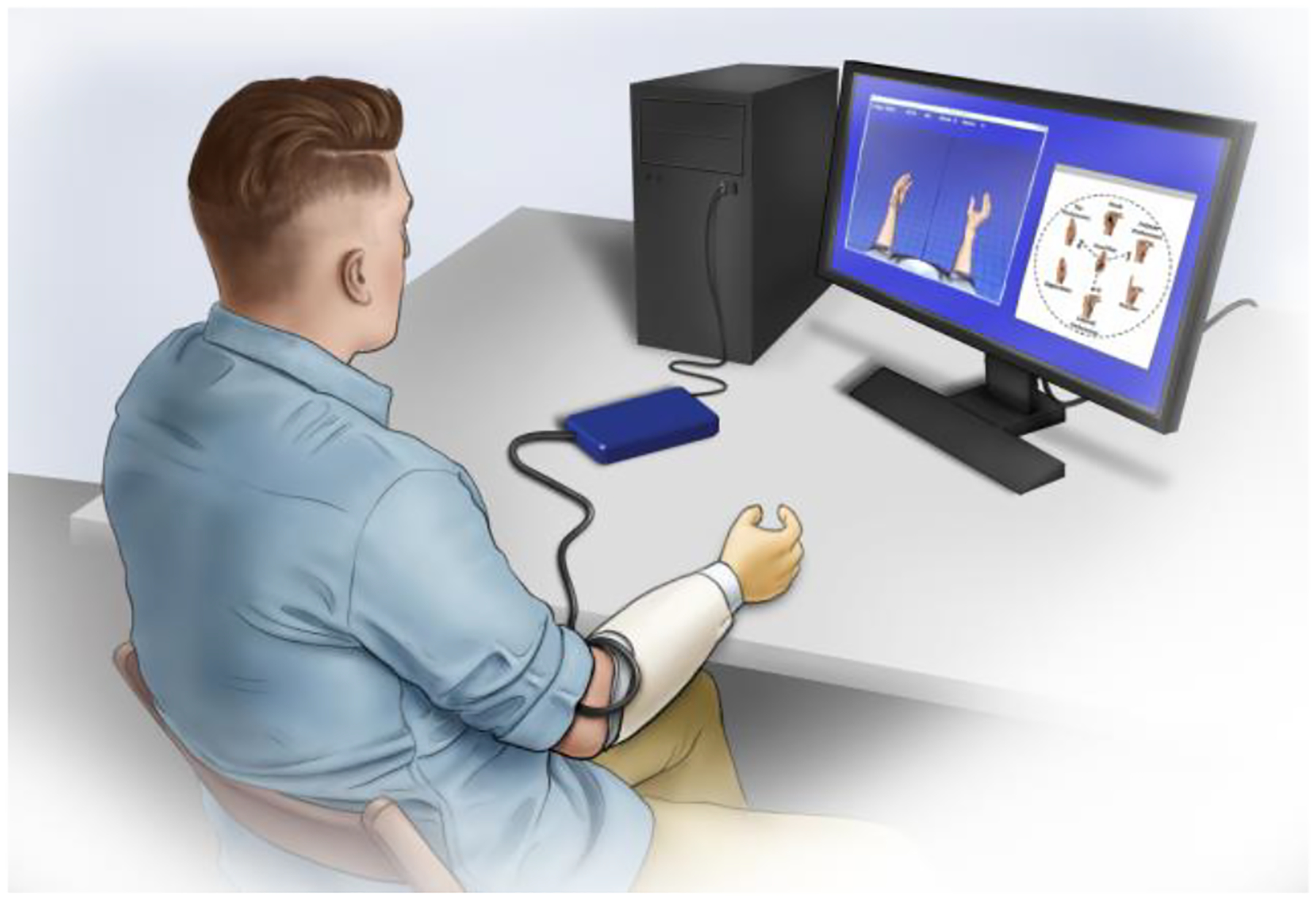
Experimental testing setup including subject with chronically-implanted EMG sensors, data acquisition system, and virtual prosthetic hand interface.

Virtual Reality (VR) target matching tasks are used as a proxy measurement for prosthesis Activity-of-Daily-Living (ADL) capabilities as VR task and ADL performance are well-correlated [30]. At its core, target matching tasks are a modified Fitts Law task [31] which describes a user’s ability to control an actuator system. During experiments, subjects were seated at a 1m distance from a computer screen and iEMG was sampled through a Ripple Grapevine Neural Interface Processor system with 15–350Hz band-pass filters at 2kHz (Fig. 1). Only the mean-absolute-value (mABS) iEMG feature was extracted using a 200ms window, updated every 50ms. The screen presented users with target postures using the VR display [15]. The VR display shows two hands with movable joints, one of which is a target and the other of which is under the user’s control. The user is also presented with a display of the Postural Control domain [24], with a red ‘X’ marking the target hand grasp. Both displays are controlled with a custom Simulink model. The user can move the controllable hand using an EMG decoder. This hand’s position is also marked with a green ‘O’ in the Postural Control domain. To match a target, the user must guide the VR hand so that every DoA is within a 15% range-of-motion window of the target posture and then remain in that window for a continuous second. The task has a 30 second time limit.

### C. Target Sets

Target sets were built separately for 2, 3, and 4-DoA tasks. The 2-DoA targets only contained hand grasps where the 1) index finger, 2) middle-ring-little fingers, 3) thumb, and 4) thumb abduction are varied to create functional postures matching the capabilities of the DEKA prosthetic limb, a 4-DoF task. The 3-DoA targets included 2-DoA hand grasps and either the flexion/extension or pronation/supination of the wrist, a 5-DoF task. The 4-DoA targets included 2-DoA hand grasps, wrist flexion/extension, and pronation/supination, a 6-DoF task. These postures mimic the capabilities of the DEKA prosthesis. Subjects were given as much rest time as they wanted between batches. Targets were built to evenly sample the acquisition of grasps, wrist positions, and both simultaneously, as previously described [16]. Grasp targets were at the end of range-of-motion; wrist position targets were either at a default orientation (full supination and slight wrist flexion, allowing maximal view of the grasp), or set randomly. Targets were interleaved with returns to a neutral posture. The DoA which were not evaluated were manually locked to the neutral hand orientation.

### D. Experimental Design

Hypotheses were evaluated in two experiments. Experiment One evaluated only the DC-PC iEMG system with both subjects. The second experiment evaluated both DC-PC and LI-PC iEMG systems with Subject S8. Both experiments evaluated performance in target-matching tasks in 2, 3, and 4-DoA cases. S6 4-DoA cases were not conducted due to an insufficient number of iEMG channels.

### E. Experiment One (DC-PC only)

**Figure 2–:**
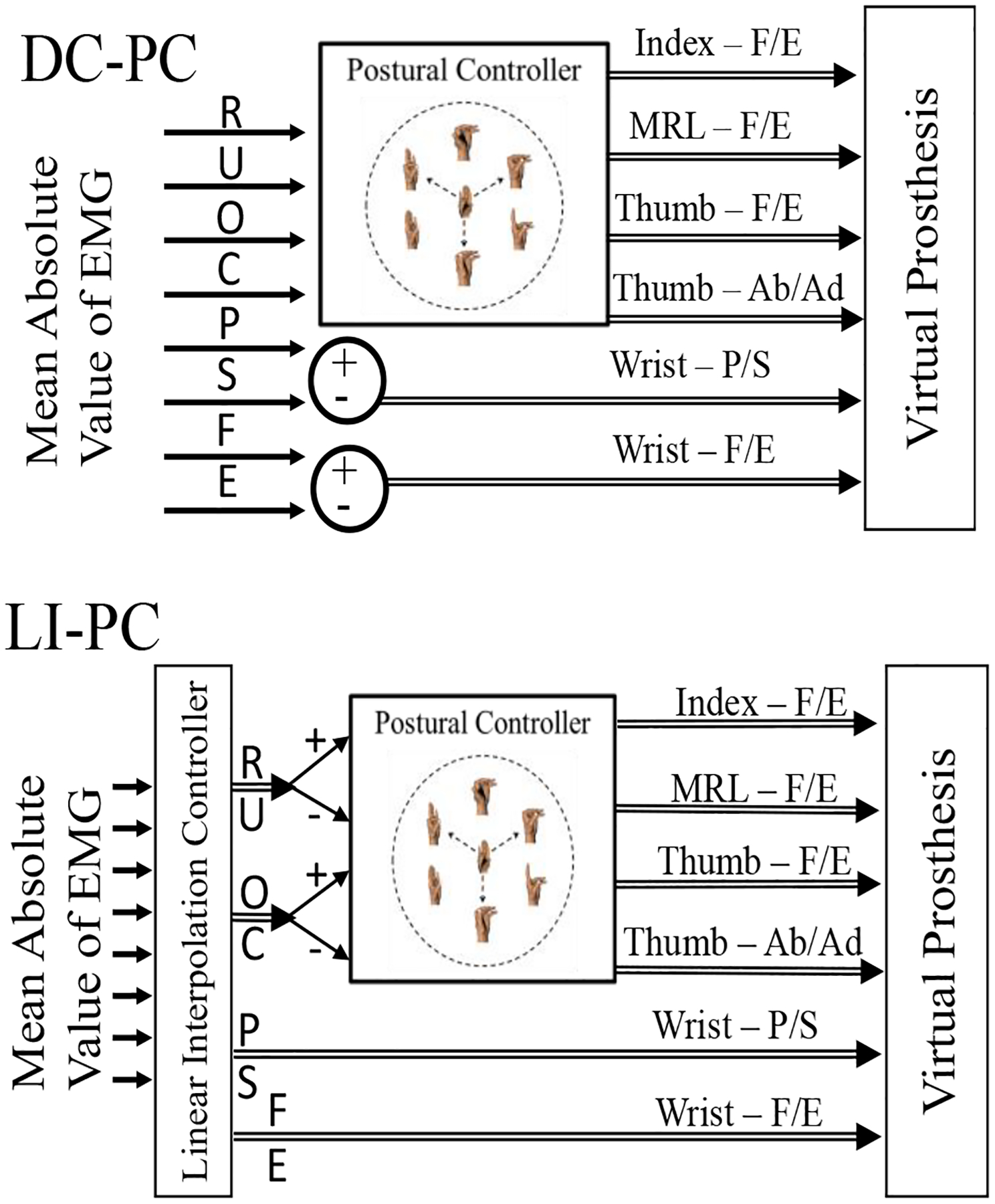
Diagram of the DC-PC and LI-PC algorithms. mABS features are extracted from ciEMG and mapped using DC or LI to estimate user’s intended movements in wrist Radial/Ulnar (R,U) deviation, hand Open/Close (O,C), wrist Pronation/Supination (P,S), and wrist Flexion/Extension (F,E) directions. These measures of intent then control the virtual prosthesis: the PC algorithm sets the hand grasp and wrist DoF are controlled in an agonist-antagonist manner (DC) or directly (LI).

Experiment One uses the DC-PC system (Fig. 2 top) which maps a small number of DoA to hand postures requiring many DoF. Past DC-PC work directly mapped sEMG signals to cursor movement directions on a 2-dimensional domain. Cursor location determined the hand grasp by interpolating pre-set grasps. In this study, iEMG signals were used and the edges of the PC domain corresponded to the grasps of the DEKA prosthesis, with the domain center corresponding to a neutral hand posture (Fig. 2). These postures are created by combining 4-DoF (index finger, coupled middle/ring/little fingers, thumb flexion/extension; and thumb ab/adduction). The PC algorithm thus maps a 2-D cursor location, modulated by a 2-DoA controller, to 4-DoF hand postures. Rather than having the 2-DoA feedforward controller output simply correspond to cursor X/Y coordinate transformations, a more intuitive mapping was chosen. Four cursor movement directions were set: three directions, 120 degrees apart, led to the Tip Prehension, Palmar Prehension, and Lateral Prehension grasps; the fourth ‘direction’ returned the grasp to a neutral hand posture. These directions were tuned to correspond, generally, to the user moving their phantom limb in a ‘Radial Deviation - R’, ‘Hand Close - C’, ‘Ulnar Deviation - U’, and ‘Hand Open - O’ directions, respectively.

Direct Control (DC) maps four EMG mABS features to the four cursor movement directions in the PC domain after applying gains and thresholds. Wrist DoA’s are controlled using remaining mABS features in an agonist-antagonist mapping. Gains, thresholds, and the mapping between iEMG channels and movement directions were set manually for every test set.

Targets for Experiment One included ten total target sets collected over two sessions per subject. All target sets were built for the 4-DoA case, included 70 targets/set, and varied between sessions. DoF’s which were not in use were simply ‘locked’ to a neutral posture, allowing many targets to be matched without movement. Of the 700 total targets, 596 were valid and the remaining 104 were excluded from analysis as they could be matched without movement.

### F. Experiment Two (DC-PC and LI-PC)

Experiment Two uses both the DC-PC system and the LI-PC system (Fig. 2 bottom). Table 1 shows iEMG-DoA mapping for each DC-PC session in Experiment Two. The mapping was determined by using the most physiologically appropriate available muscle site for each DoA. The Linear Interpolation feedforward controller (LI) uses EMG signals to predict a user’s intended control signal and is described in past work [16]. Briefly, LI is constructed first by limiting EMG from sampled user movements to steady state and identifying the pattern of EMG activity for each movement. These patterns are then normalized to fall on a single hyperplane. A triangulated irregular network on this hyperplane is then created by treating the normalized EMG patterns as vertices and using Delaunay triangulation. These steps effectively partition EMG feature space into regions emanating from the origin where each region is bounded by N EMG patterns, where N is the number of EMG channels. Online user signals are decoded by linear interpolation on this triangulated network: the partition that the signal appears in is identified and the movements that bound the partition are linearly interpolated to determine the user’s movement. Whereas typical pattern recognition algorithms only allow movements in pre-defined directions, LI simultaneously sets the direction and speed of the prosthesis and has been shown to allow simultaneous, proportional, and continuous control of up to 4-DoA. Training movements included all single and paired DoF that involved wrist pronation/supination, flexion/extension, radial/ulnar deviation, and a hand open/close DoF. The same VR set used in evaluation was used for training data collection. Training data was collected 9 months prior to the real-time evaluation within this study and provided stable performance over months of time [16]. User intent corresponding to Radial/Ulnar deviation and Hand Open/Close movements is passed to the PC algorithm to determine hand movement. Decoded wrist command signals control the virtual prosthetic’s wrist directly. The LI-PC controller was built separately for each experimental DoA condition. Experiment Two involved only Subject S8 and used 2, 3, and 4-DoA target sets. Targets were presented in 4 batches of 15 for a total of 60 targets, except the 6 DoA case which included 3 batches of 16 and 1 batch of 14, for 62 total targets. Errors in manually ‘locking’ unused DoA invalidated 11 of 488 evaluated targets by either making them un-reachable or reachable without movement. These 11 targets were excluded from evaluation and did not affect study results given their small number.

### G. Metrics and Statistics

Four outcome measures were used to describe the subjects’ performances. Match Rate (MR, %) is the percentage of targets matched. Time-To-Target (TT, seconds) is the average time needed to match a target posture, excluding the one-second dwell time. Path Efficiency (PE, %) measures the directness of the path taken in DoF-space to reach the target posture. As an example, a 33% PE indicates that the virtual hand traveled three times further than a straight-line path to the target. User Exertion (UE, sec*V/V) measures the effort required by the subject to achieve the target. This is found by normalizing iEMG features to the peak values observed in the training data for the LI controller (a self-selected ‘medium level of effort’) and integrating these values over the time needed to match each target [23]. These metrics describe the utility (MR, TT) and burden (PE, UE) of the controller and are a proxy for the performance with a prosthetic limb system.

Statistical analyses were performed at α = 0.05 using Matlab (The MathWorks Inc., Natick, Massachusetts). For Experiment One, S6 TT and PE were compared with unpaired t-tests. S8 TT/PE were compared with a one-way ANOVA followed by unpaired t-tests. MR for S6 and S8 was compared with a Fisher’s exact test. Bonferonni corrections were applied within each metric and session. For Experiment Two, LI-PC and DC-PC TT/PE/UE were compared with paired t-tests and MR were compared with Fisher’s exact test. As results are expected to be highly correlated, Bonferonni corrections were not applied. The 3-DoA cases were further compared post-hoc with unpaired t-tests and fisher’s exact test with Bonferonni corrections.

## III. Results

### A. Real-time, simultaneous, and proportional 6-DoF control

We verified during the 4-DoA sessions that the subject could control the 6-DoF virtual hand and wrist system in a simultaneous fashion using both control algorithms. EMG signals indicated concurrent activity, and the paths in joint space indicated simultaneous, proportional movement. The subjects were not constrained to drive the DoFs in a sequential. Their behavior showed simultaneous, proportional control of all 4-DoA’s during the tasks. The ability to control all DoA’s in a simultaneous manner, rather than sequential, is an advancement in the control of high degree of freedom prosthetic devices.

### B. DC-PC performance decreases as DoA increase

**Figure 3.**
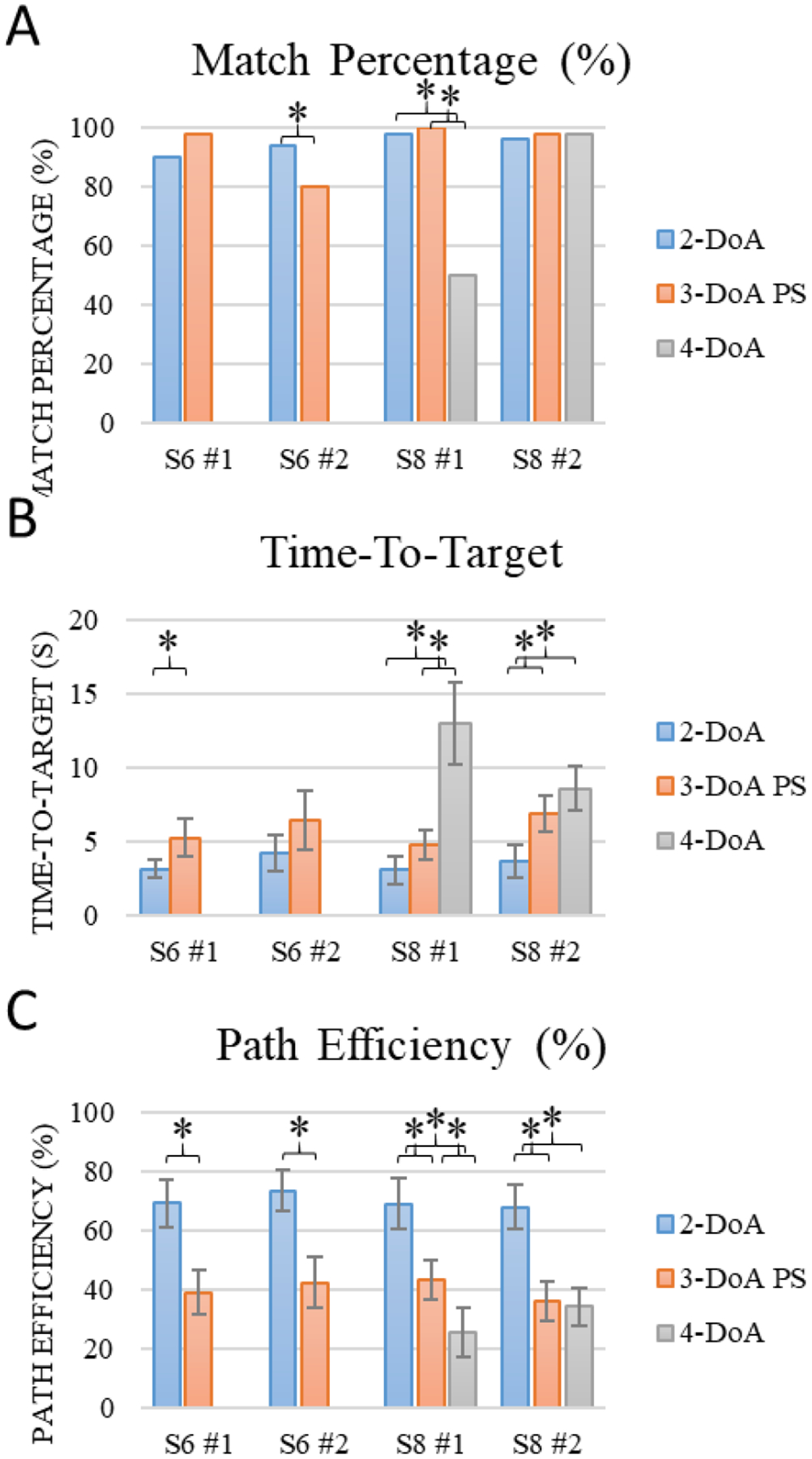
Experiment One. DC-PC evaluations with both subjects, two days per subject. DoA conditions are split by session and presented chronologically. Identical targets are presented across DoA conditions and unused DoF’s are simply locked. Values are shown as mean and 95% CI. * denotes significant difference.

Results from Experiment One are shown in Fig. 3. Results from both subjects across two sequential experimental days are presented. Substantial reductions in MR, TT, and PE performance were observed as DoA increased. In no cases did a performance metric substantially improve as DoA increased. The TT and PE metrics showed significant differences between the 2, 3, and 4-DoA tasks across nearly all subjects and experimental days. These results highlight that both subjects could achieve 4-DoA, 6-DoF control using the DC-PC algorithm but with diminishing ability compared to the lower DoA tasks.

### C. LI-PC improves metrics versus DC-PC at higher DoA

**Figure 4.**
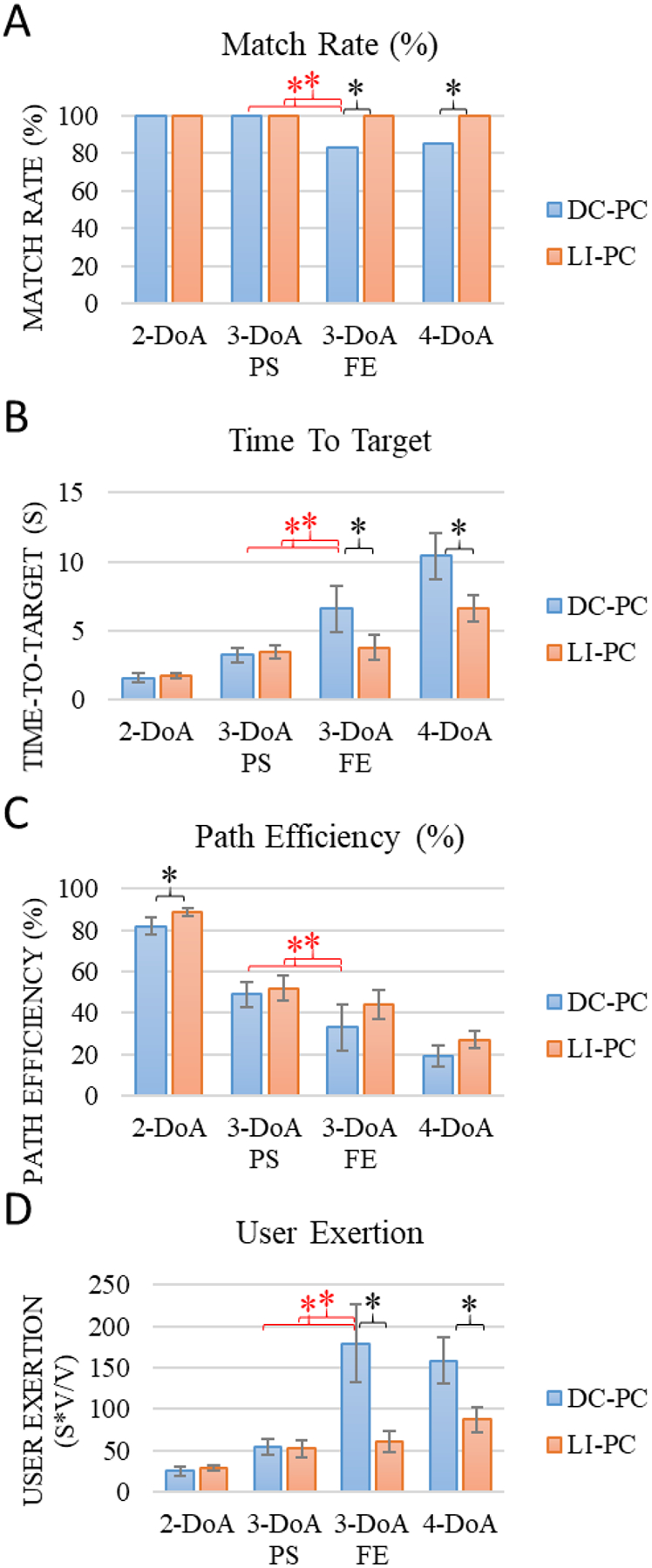
DC-PC and LI-PC systems with ciEMG were compared with subject S8. The figure presents 2-DoA, 3-DoA PS, 3-DoA FE, and 4-DoA conditions. Only targets achievable by each DoA condition are presented in each condition and vary across experimental conditions. Values shown as mean with 95% CI. Black asterisks denote significance in pairwise comparisons (*Results: C*); red asterisks denote significance in further un-paired post-hoc comparisons of 3-DoA cases (*Results: D*)

The results of Experiment Two depict the comparison of DC-PC and LI-PC (Fig. 4). In comparing DC-PC and LI-PC, performance differed significantly in MP, TT, and UE for the 3-DoA F/E and 4-DoA cases. These findings indicate that user command signal decoding can improve the DC-PC system using with iEMG. In particular, the LI-PC controller is more effective (MP, TT) and less burdensome (UE) than DC-PC at higher DoA. The benefit of the LI-PC controller is consistently seen across the higher DoA results for MR and TT metrics which indicate the improved utility of the LI-PC compared to the DC-PC. Furthermore, the dramatic reduction in UE during the higher DoA tasks demonstrates the reduction in burden on the subject to control the limb in a simultaneous fashion. The lack of difference between the two control strategies at lower DoA implies that iEMG signals can be readily interfaced with existing direct control strategies for prosthetic limb systems with 2 or fewer DoA’s.

### D. Further analysis of 3 DoA P/S and F/E trials

**Figure 5–:**
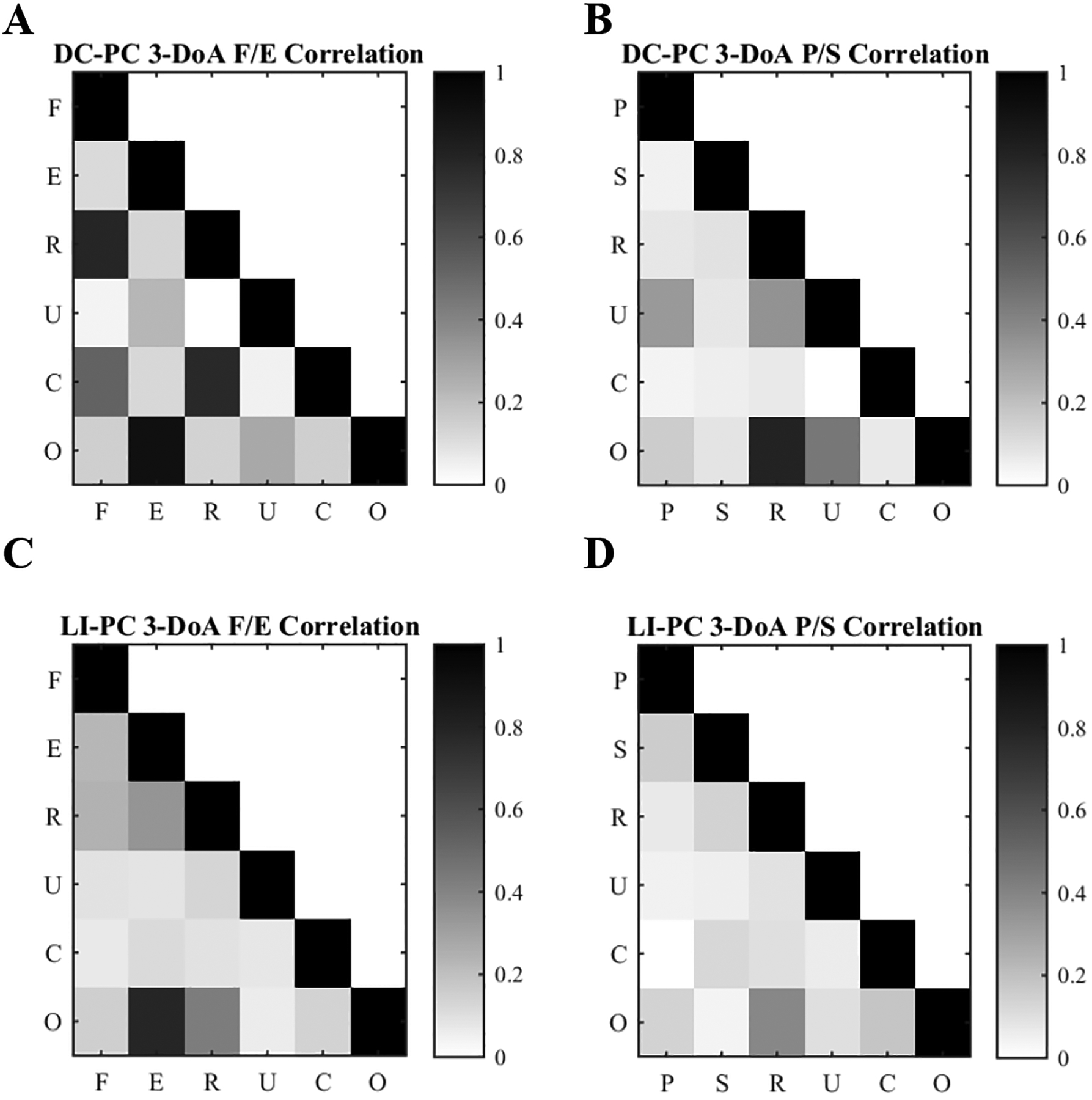
Correlations in decoded user command signals were identified for each 3-DoA case in Experiment Two. F – Wrist Flexion; E – Wrist Extension; R – Wrist Radial Deviation (Tip Prehension grasp); U – Ulnar Deviation (Lateral Prehension grasp); C – Hand Close (Palmar Prehension Grasp); O – Hand Open (No Grasp); P – Wrist Pronation; S – Wrist Supination. Note: Dark colors show highly correlated user command signals which inhibit independent DoA control.

The 3-DoA F/E and P/S cases were further analyzed due to the curious fact that the complexity was equal, but the performance was significantly different when controlling the F/S DoA compared to the P/S DoA (Fig. 4, red). The DC-PC 3 DoA F/E case had significantly worse MP, TT, and UE than all other 3-DoA cases and worse PE than both 3-DoA P/S cases. To further investigate 3-DoA cases, a correlation analysis was performed across decoded command signals; these are shown in heat-maps in Fig. 5 for all 3-DoA cases. High correlation coefficients (dark cells in Fig. 5) indicate that prosthesis command signals are correlated which could inhibit target matching. The DC-PC 3-DoE F/E case (Fig. 5a) had more highly correlated control output signals than other 3-DoA controllers (Fig. 5b–d). LI-PC cases have lower decoded command signal correlation than DC-PC cases. We hypothesize that the more highly correlated F/E command signals caused a reduction in performance compared to the P/S trials. The reduction of correlation across command signals is critical for high DoA control when using iEMG or sEMG and is achieved more readily with the LI-PC controller.

## IV. Discussion

### A. Progress on Postural Control for High DoA Systems

The PC algorithm was previously shown to provide benefit in the control of multi-functional prosthetic hands when using multiple grasps [22]–[25]. This work describes the first use of the PC algorithm with iEMG. It also presents the first case where the PC algorithm was used in a 4-DoA HMI that included simultaneous control of grasps, wrist pronation/supination and wrist flexion/extension in a virtual limb system. This work demonstrates Postural Control to be a viable post-processing step for arbitrary decoding methods, which hypothetically allows any machine learning approach to control grasps rather than individual DoF. This is valuable in the context of enabling better clinical use of complex prosthetic limbs such as the Luke Arm from DEKA Research and Development Corporation.

### B. iEMG Enables High-DoF Direct Control with Little Tuning

One surprising finding in this study was that subject S8 achieved good 4-DoA / 6-DoF control with the DC-PC system. Past work has shown that direct control can allow up to 2-DoA control with fine-wire EMG recordings in a position control manner [19]. Indeed, DC is expected to be limited to 2-DoA as there are believed to be at most four independent surface EMG sites on a residual limb [32] and using additional sites provides no benefit due to extensive crosstalk [33]. Machine learning is the usual method for increasing control to 3-DoA sEMG classifiers [8]. iEMG provides additional independent EMG sites, increases signal specificity, and reduces co-activity [15] [20], [21], [34]. Leveraging iEMG has allowed the development of simultaneous, continuous, and proportional 3-DoA and 4-DoA controllers. However, these controllers still use subject training data for regression [20], k-nearest-neighbor [34], or interpolation [16] methods. It is therefore promising to observe 4-DoA simultaneous, continuous, and proportional control arising from direct control with iEMG after only a short tuning period.

Compared to past work, using iEMG appears to improve control both in low and high DoA cases. For example, the Path Efficiency of the 2-DoA virtual hand in this study was 89% with the LI-PC system and 82% with DC-PC. This is greater than previous work involving the 2-DoA hand engaging 3, 4, and 12-sites of surface EMG which allowed a 69% PE [24]. Intramuscular EMG thus seems to improve subjects’ ability to complete this experimental task in an efficient manner and reduce the burden on the subject.

### C. Front-end processing of iEMG augments control

Study results indicate that the DC-PC interface is sufficient for some degree of 4-DoA/6-DoF control; nonetheless, performance improved in LI-PC cases in conditions that involved wrist flexion/extension. This was coupled with a reduction in user command signal correlation and substantial reductions in user exertion. These results imply that there is benefit in using front-end processing of the iEMG signals to improve independence of the iEMG signals and thereby improve prosthetic control. This is not surprising, as following motor synergy theory [35] users are expected to intuitively command muscles in groups rather than independently. In result, co-activity across EMG signals will be present even when using iEMG. These findings are significant as the field of upper limb prosthetic control develops implantable technology that will still be recording co-activity across EMG sites. A front-end processing algorithm like the LI presented here will be beneficial to maintain command signal independence.

### D. Study Limitations

This study has limitations across both the controllers tested and study design. First, the PC algorithm is not an intuitive mapping of the iEMG control signals to the DoFs of the virtual limb. The algorithm can map arbitrary signals (e.g. EMG channels in the DC case, decoded intent the LI case) to hand grasps. However, the movements users make with their phantom limb will not inherently line up with the grasp changes undertaken by the prosthesis. Fortunately, it appears that these mappings can be learned rapidly. The study design has several constraints that limit the generalization of the results. The number of subjects involved was limited to two. To our knowledge, only two subjects have the implanted hardware necessary to participate in the study and both were involved. This is not unusual for case series involving hardware implanted under an Investigation Device Exemption. Furthermore, effort was made to collect a large volume of data totaling over a thousand virtual tasks measured. The volume of data ensures statistically relevant comparisons among the small number of subjects. The data was collected over a limited period due to constraints on subject time. Most results (Results Sections C-D) involved only one subject during one session. However, the exploration of Direct Control with PC space involved two subjects, with two sessions each spaced several months apart (Results Sections A-B). The drawbacks of chronically implanted EMG include intensive surgical procedures and recovery time, longitudinal observations to monitor infection, and the risk of device failure.

## Figures and Tables

**TABLE I.** MAPPING OF ciEMG TO MOVEMENT DIRECTION IN EXPERIMENT TWO

Movement Direction	Muscle Used			
(U) Lateral Prehension	ECU	ECU	ECU	ECU
(R) Tip Prehension	ECRL	ECRL	FCR	FCR
(C) Palmar Prehension	FCU	FCU	FCU	FCU
(O) Hand Open	ED	ED	ED	ED
Wrist Extension			ECRL	ECRL
Wrist Flexion			Pronator	Pronator
Pronation		Pronator		FDS
Supination		Supinator		Supinator
	2-DoA	3-DoA P/S	3-DoA F/E	4-DoA

## Clinical Relevance

This work demonstrates a novel method of providing simultaneous control of a 6 DoF virtual wrist and hand prosthesis.
